# Probing the chemical ‘reactome’ with high-throughput experimentation data

**DOI:** 10.1038/s41557-023-01393-w

**Published:** 2024-01-02

**Authors:** Emma King-Smith, Simon Berritt, Louise Bernier, Xinjun Hou, Jacquelyn L. Klug-McLeod, Jason Mustakis, Neal W. Sach, Joseph W. Tucker, Qingyi Yang, Roger M. Howard, Alpha A. Lee

**Affiliations:** 1 Cavendish Laboratory, University of Cambridge, Cambridge, UK; 2 Pfizer Research and Development, Groton, CT USA; 3 Pfizer Research and Development, La Jolla, CA USA; 4 Pfizer Research and Development, Cambridge, MA USA

**Keywords:** Cheminformatics, Organic chemistry

## Abstract

High-throughput experimentation (HTE) has the potential to improve our understanding of organic chemistry by systematically interrogating reactivity across diverse chemical spaces. Notable bottlenecks include few publicly available large-scale datasets and the need for facile interpretation of these data’s hidden chemical insights. Here we report the development of a high-throughput experimentation analyser, a robust and statistically rigorous framework, which is applicable to any HTE dataset regardless of size, scope or target reaction outcome, which yields interpretable correlations between starting material(s), reagents and outcomes. We improve the HTE data landscape with the disclosure of 39,000+ previously proprietary HTE reactions that cover a breadth of chemistry, including cross-coupling reactions and chiral salt resolutions. The high-throughput experimentation analyser was validated on cross-coupling and hydrogenation datasets, showcasing the elucidation of statistically significant hidden relationships between reaction components and outcomes, as well as highlighting areas of dataset bias and the specific reaction spaces that necessitate further investigation.

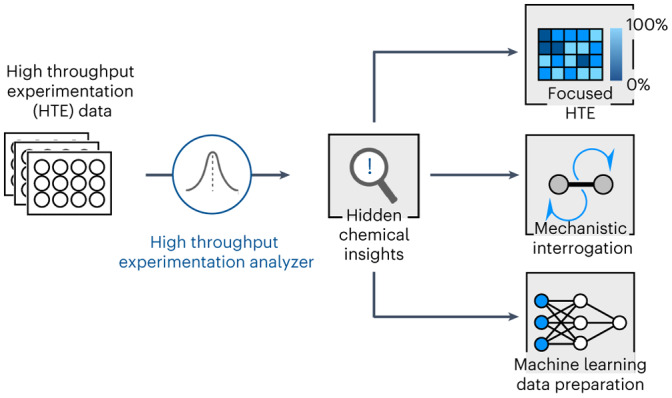

## Abstract (web summary)

High-throughput experimentation (HTE) has great utility for chemical synthesis. However, robust interpretation of high-throughput data remains a challenge. Now, a flexible analyser has been developed on the basis of a machine learning-statistical analysis framework, which can reveal hidden chemical insights from historical HTE data of varying scopes, sizes and biases.

## Main

Data-driven chemistry has seen immense strides in recent years, especially in yield and enantioselectivity prediction^1–4^. One major contributing factor to this is the adoption of high-throughput experimentation (HTE) data in chemical synthesis^5–8^. Collections of ‘real-world’ HTE data have several beneficial features. They probably have sampled the reaction space that is of direct interest to the field and cover a broad range of substrates and reaction types, ensuring that data-driven findings are relevant^9^. Valuable negative data is also present^10^. Additionally, the data will probably have been gathered in a manner that enables future HTE-guided synthesis, aiding the translatability of the findings. This approach is, however, not without its challenges. Yield calculations are often derived from the uncalibrated ratio of ultraviolet absorbances, which assumes that the species have similar ultraviolet extinction coefficients and makes this measurement more qualitative than quantitative nuclear magnetic resonance spectroscopy or isolated yield determinations. The presence or absence of byproducts may also be somewhat obscured (see Supplementary Information for a full discussion on yield determination on HTE datasets). Moreover, datasets may be subject to biases in reactant and reaction condition selection and have regions of substantial data sparsity.

**Fig. 1:**
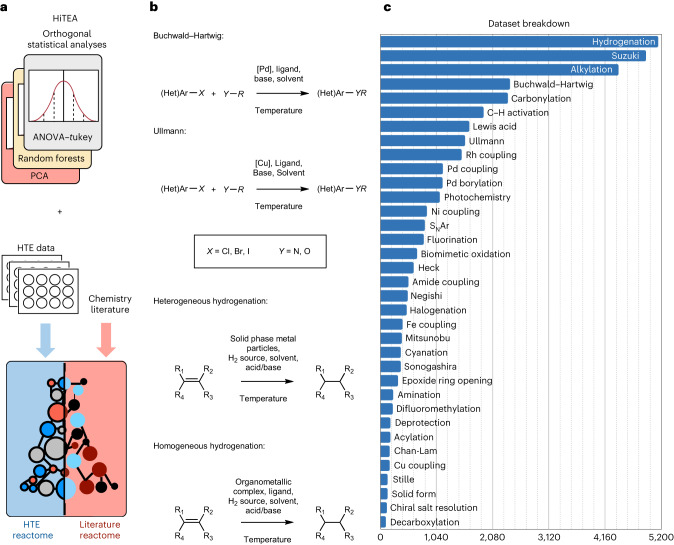
Overview of the HTE dataset and framework. **a**, Overview of HiTEA and its analysis is shown. Comparison of the literature reactome with HiTEA reactome will reveal support for our mechanistic conclusions (agreement of reactomes) or reveal areas of bias/unusual chemical phenomenon (disagreement of reactomes). **b**, Abstracted representations of the four reaction classes analysed by HiTEA in this publication are shown. **c**, Breakdown of the HTE dataset by reaction class is shown.

Despite these known challenges with HTE data, little work has been done to investigate the inherent structure and biases of these datasets^11^. A statistically robust methodology that can be applied to any HTE dataset to draw out hidden chemical insights is fundamental to driving forward data-driven chemistry. It is important to note that that this statistical framework was not envisioned to predict or generalize any specific reaction property (yield, selectivity, optimal conditions and so on), but to provide a far more fundamental analysis: what are the chemical insights within a dataset? From these conclusions, we can begin to understand (1) what are statistically important factors that drive good or bad outcomes and (2) what this data will teach an artificial intelligence (AI) model. Finally, comparison of the chemical insights embedded within the HTE data, what we dub the ‘HTE reactome’, to the chemical insights drawn from the literature, the ‘literature’s reactome’, may (1) provide further evidence to support the mechanistic hypotheses (agreement of the reactomes), (2) reveal bias within the dataset which limits its usefulness or (3) reveal subtle correlations that may lead to refinement of our chemical understanding (disagreement of the reactomes) (Fig. 1a). In this Article, ‘literature’ is defined as information from open-source chemistry databases and published literature in peer-reviewed journals that excludes HTE data.

To create such a methodology, a high-throughput experimentation analyser (HiTEA) was developed, which can deduce the reactome of any HTE dataset. While common chemistry datasets such as the CAS^12^, Reaxys^13^, USPTO, Pistachio or the Open Reaction Database have impressive coverage, it was a concern that the high level of overlap between their reactions and literature data would shape these datasets’ reactomes to be indistinguishable to literature reactomes^14,15^. This would make it difficult to explore the discrepancies between the data and the literature reactomes, something that is probably possible when utilizing HTE datasets and a fundamental feature of HiTEA that we wished to investigate. Thus, a HiTEA analysis was performed on a ground-breaking release of 10 years of historical medicinal chemistry HTE data. It is an unprecedently large dataset, acquired over 10+ years and spans a wide range of reaction classes (Fig. 1c). Within it are over 39,000 reactions conditions spanning over 350 target products. The reactions are split across numerous classes, ranging from thousands of reactions to tens of reactions, whose reactants and reagents may be over-represented or under-represented. These challenges highlight the necessity of statistical analyses, which can understand the data even in these skewed environments. HiTEA’s analysis of several classic reaction types reveal some notable biases as well as some unexpected findings which warrant further investigation.

## HiTEA: statistical analysis framework for HTE

The HiTEA methodology is centred around three orthogonal statistical analysis frameworks, random forests, *Z*-score analysis of variance (ANOVA–Tukey) and principal component analysis (PCA). Each framework answers one of the following questions: which variable(s) are important? namely, random forests^16^; what are the statistically significant best-in-class/worst-in-class reagents? namely, the *Z*-score–ANOVA–Tukey^17–20^; and how do those best-in-class/worst-in-class reagents populate the chemical space? namely, PCA^21,22^. Notably, this combination of statistical analysis makes no assumption about the underlying data structure. For example, relationships can be non-linear or even discontinuous, the data does not need to be the full combinatorial cross of all reagents with all reactants, an important feature when considering the sparse nature of chemistry datasets, and smaller datasets are just as feasible as larger datasets. The synergy between these three branches of HiTEA paint a comprehensible understanding of a dataset’s reactome, allowing for facile identification of hidden chemical insights. To highlight the flexibility and versatility of HiTEA, we analyse datasets that span upwards of 3,000 reactions across a broad range of substrates to datasets that are just over 1,000 reactions with a narrower substrate scope.

## Which variables are most important?

Intuitively, some reactions are more sensitive to certain variables than others. Cross couplings are highly sensitive to the metal and its ligand, but generally less sensitive to the identity of the solvent^23,24^. The relative variable importance is critical to understanding the chemistry insights that are present in the reactome. Note that importance can be positively correlated or negatively correlated with reaction outcome.

When investigating variable importance, two techniques come to mind as versatile and broadly applicable: random forests and multi-linear regressions. Both have yielded impressive results in chemistry and other field; however, for HiTEA we chose to utilize random forests^4,16,25,26^. Unlike multi-linear regression, random forests do not stipulate that one’s data must be linear, and thus obviate the need for linearization (and ideally normalization). Given the non-linearity of the data, we hypothesized that random forests would yield more accurate variable importances. In general, moderate-to-good out of bag accuracy of reaction outcome from a random forest with standard hyperparameters was observed (Supplementary Table 1), with some noted exceptions (taking dataset to HiTEA sections), correlating with poorer mechanistic insights of the reaction class overall. To assess the confidence of the variable importance, ANOVA was performed on each dataset subclass with statistical significance of the variables set at *P* = 0.05.

## What are the best- and worst-in-class reagents?

It is known that there are privileged reagents that perform well across the board for a multitude of reactions, and there are those whose utility is narrow. Identifying the best- and worst-in-class reagents is therefore key to understanding a reactome. However, detangling the impact of a reagent from the inherent reactivity of the reactant(s) is challenging. We chose to compare relative yields that had been normalized through *Z*-scores, a technique that has shown promise in analysis of HTE data^17,27^. Notably, this framework allows for other target reaction outcomes to be used such as diastereoselectivity/enantioselectivity. ANOVA on the normalized target reaction outcome reveals the broad variables (solvent, base, catalyst system and temperature, and so on) that are statistically relevant for that reaction outcome^17–19^. Tukey’s honest significant difference test is then used to identify the outliers in each statistically significant variable, which are then ranked by average *Z*-score to provide the best- and worst-in-class reagents^20^.

## How do the reagents populate the chemical space?

A visualization of the best- and worst-in-class reagents is useful to contextualize the scope of the dataset and therefore the extent of the reactome. The selection bias of reagents and clustering of high and low performing reagents can be easily interpreted. While numerous techniques for dimensionality reduction and visualization of high-dimensional space are known, we chose to use PCA as its utility has been widely documented and numerous reliable user-friendly implementations exist^28,29^. Additionally, PCA is more interpretable than uniform manifold approximation and projection or t-distributed stochastic neighbour embedding, whose non-linearity necessitate warping the high-dimensional shape of the data during projection; the *xy* axes of projection lose the easy interpretability of highest variance (*x* axis)/second highest variance (*y* axis) that is fundamental to PCA^30,31^.

## Taking the dataset to HiTEA

To test HiTEA, four distinct reactomes were chosen to be explored. These reactomes were widely used reaction classes: Buchwald–Hartwig couplings, Ullmann couplings, heterogeneous hydrogenations and homogeneous hydrogenations (Fig. 1b). From the generated reactomes, careful analysis of HiTEA’s variable importances, statistically relevant best-/worst-in-class bases and catalysts, and ligand distribution was performed, concluding with tailored recommendations for further exploration. This analysis was also performed on temporally segregated data and data with their 0% yielding reactions removed, to mimic a dataset that would be more likely found in literature sources. Generally, temporal analysis appeared to be better correlated with the series of individual substrates screened over time than the evolving screen designs themselves. Despite the noticeable drift in best and worst reagents over time, we believe that inclusion of older data points may be beneficial. First, it typically expands the substrate space investigated, which is beneficial when investigating a reaction class overall. It also allows HiTEA and the user to more clearly distinguish the highly versatile reagents, which can be observed as those that were excellent performers over the entire course of their usage. The removal of 0% yielding reactions lead to a far poorer understanding of the reaction class overall (Supplementary Figs. 1–8). The disappearance of the worst-in-class reagents and catalysts was expected; however, best-in-class conditions also disappeared. This result highlighted the value of 0% and lower-yielding data in the disclosure of all datasets.

## Buchwald–Hartwig couplings

Buchwald–Hartwig couplings are a fundamental reaction in medicinal and process chemistry^32^. The dependence of yield upon ligand electronic and sterics is well reflected in this dataset; it is diverse in catalysts and ligands, but less diverse in coupling partners. This was the largest reactome we analysed consisting of ~3,000 reactions.

**Fig. 2:**
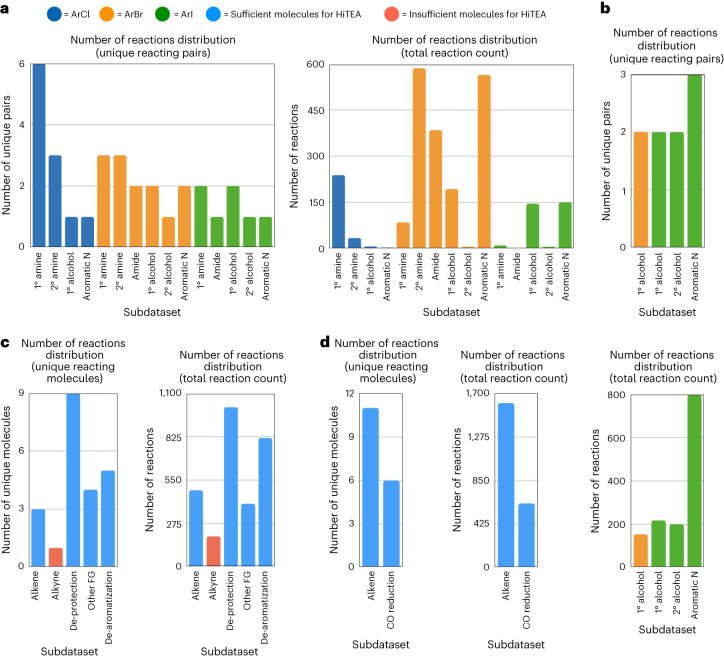
Unique reacting pairs/molecules for each reaction class. **a**–**d**, Buchwald–Hartwig dataset (**a**), Ullmann dataset (**b**), heterogeneous hydrogenation dataset (**c**) and homogeneous hydrogenation dataset (**d**) are shown. FG, functional group.

Diversity wise, the dataset contained 31 unique halides and 32 unique nucleophiles, encompassing amine, amide, aromatic nitrogen and alcohol nucleophiles, and 29 unique reacting halide–nucleophile pairs. Interestingly, the nucleophiles were less diverse than the aryl halides, owing to the nature of the ongoing campaigns at the time (Supplementary Fig. 9). It was also found that aryl bromides made up the majority of the reactions, both in number of unique reacting pairs and total number of reactions (Fig. 2a). It was expected that HiTEA on the Buchwald–Hartwig dataset without accommodating for this over-representation would reveal an HTE reactome predominantly centred around aryl bromide couplings. Indeed, HiTEA credits high variable importance to BrettPhos Pd G1 (Supplementary Fig. 10). This is clearly not in agreement with the literature’s reactome, in which many ligands show equal or better general performance to BrettPhos^11,23,24,33^. Thus it was hypothesized that a more nuanced analysis would arise if HiTEA was be applied to subdatasets (that is, ArBr + 1° amine, ArCl + 1° amine, and so on), to determine their subreactomes. Subdatasets with more than 80 reactions and two or more unique reacting pairs were analysed, as these subreactomes were more likely to be differentiated from their literature chemical reactomes.

**Fig. 3:**
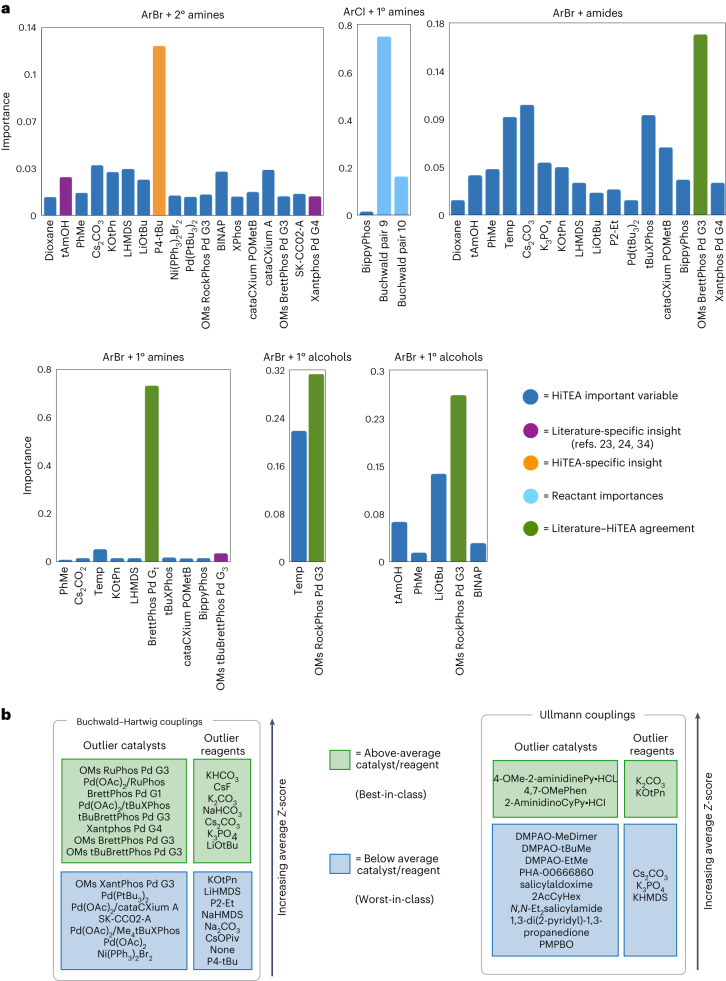
HiTEA analysis of the Buchwald–Hartwig dataset. HiTEA/literature-specific variable importances agreement between the literature and HiTEA variable importances is highlighted. Acronym structures can be found in in Supplementary Fig. 11. Temp, reaction temperature. **a**, Variable importances are shown and unless otherwise specified, the metal source for the ligand is Pd(OAc)_2_. Where appropriate, reactant importances are shown. **b**, Statistically significant best-/worst-in-class catalysts and reagents are shown and unless otherwise specified, CuI is the copper source for the Ullmann couplings.

With ArBr + 1° amines (three unique reacting pairs), the literature precedent suggests a high dependence on bulky biaryl phosphine ligands^23^, which can inhibit the unproductive β-hydride elimination pathway and prioritize the reductive elimination, will be observed. BrettPhos ligands were expected to be dominant in the reactome’s variable importances^34^, and indeed, we see that BrettPhos Pd G1 is by far the most important variable for this subdataset. Surprisingly, the even bulkier *t*-BuBrettPhos was not in contention for the top important variable^23,24^. The ArBr + 2° amines (three unique reacting pairs) show a negative dependence on the presence of P4-*t*-Bu, a phosphazene base, which despite known utility in cross couplings^35^, is universally bad for this subdataset. The other phosphazene base, P2-Et, is also ranked poorly by HiTEA (Fig. 3b). A recent systematic investigation of optimal standard Buchwald–Hartwig conditions noted that P2-Et underperformed other bases^9^. With ArBr and ArI + 1° alcohols (both with two unique reacting pairs), and ArBr + amides (two unique reacting pairs), ligands with rigid backbones and steric bulk that promote easier reductive elimination and prevent the deleterious K^2^-amidate complexes^24,36^ were expected to dominate, although a lower diversity of catalysts present in the variable importance analysis could be due to the lower random forest out of bag accuracy for these two reaction classes (Supplementary Table 1). For these three subdatasets, the subreactomes are in agreement with the literature’s reactome with OMs RockPhos Pd G3 and OMs BrettPhos Pd G3 highlighted in HiTEA’s analysis. Finally, we turn to ArCl + 1° amine couplings (six unique reacting pairs). Here, the literature reports electron rich ligands that allow for more facile oxidative addition of the Ar–Cl bond and bulky scaffolds that limit the known β-hydrogen elimination pathway are preferred^37,38^. However, the HTE subreactome had only Pd(OAc)_2_/BippyPhos as a variable of minor importance (Fig. 3a). Upon closer inspection, a high dependence upon substrate identity was observed, implying that for this subreactome, the most important factor is the reacting halide–nucleophile pair.

**Fig. 4:**
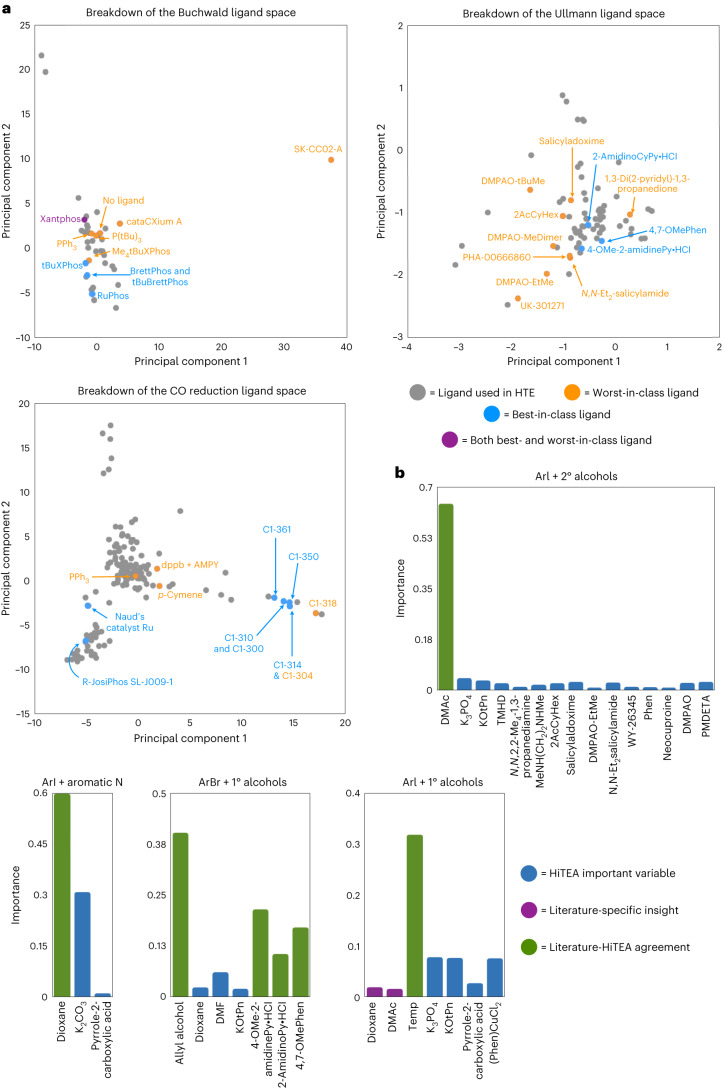
Visualization of the ligand space for all three subreactomes and in-depth analysis of Ullmann dataset. **a**, PCA ligand analysis of the Buchwald, Ullmann and CO reduction ligands is shown. **b**, HiTEA variable importance analysis of Ullmann dataset is shown. Unless otherwise specified, CuI is the copper source. HiTEA/Literature-specific variable importances agreement between the literature and HiTEA variable importances is highlighted. Acronym structures can be found in Supplementary Fig. 11.

Overall, the best-/worst-in-class catalysts fall neatly into chemical intuition for the reasons highlighted above (Fig. 3b), and gratifyingly also cluster neatly in the ligand PCA visualization (Fig. 4a). A sharp divide between best-in-class and worst-in-class ligand clustering is clear and Xantphos, the single ligand that could be either depending upon the precatalyst employed, resides away from the other ligands. Many of the subreactomes also agree with the literature’s reactome, but several areas of interest stick out. First, the ArCl + 1° amines reactome differs from the literature’s. While ArCl + 1° amine yield are somewhat dependent upon their reactants’ structures, the lack of any major ligand importance and the dominance of reactant identity suggests to us that this dataset may have some substrate selection bias. A clearer picture of ArCl + 1° amines’ reactome could be achieved with expansion of the diversity in nucleophiles screened. The second is the little importance placed upon *t*-BuBrettPhos in ArBr + 1° amine’s reactome. This may be due to the infrequent usage of *t*-BuBrettPhos when compared with the other catalysts in the subdataset. In the instances that *t*-BuBrettPhos was utilized, it was with challenging substrates (hence why it was noted as a best-in-class ligand with *z*-score–ANOVA–Tukey). In future screens, it could be advantageous to use *t*-BuBrettPhos more frequently to investigate this further.

## Ullmann couplings

In recent years, palladium-free cross couplings such as the Ullmann reaction have gained in popularity due to their cost-effectiveness^39^. Ullmann couplings, in particular, are a viable option for aryl bromide/iodide and nucleophile cross couplings. The Ullmann dataset is more modest in scope and scale than its Buchwald–Hartwig counterpart, at about half the size; however, even in this smaller space HiTEA is applicable.

Contrary to the Buchwald–Hartwig dataset, which encompassed a ‘wide but shallow’ sampling of the substrate space, the Ullmann reactions are ‘narrow but deep’ with few subdatasets but higher total number of reactions for each (Fig. 2b). The dataset contained nine unique halide–nucleophile pairs, with good diversity in both the aryl halides and the nucleophiles, albeit a limited number of each (Supplementary Fig. 12).

HiTEA revealed HTE subreactomes that readily distinguish between subtle differences in solvent. Across the board, a high importance of solvent is observed, with dependencies based on differing reactomes. For example, In the ArI + aromatic nitrogen (three unique reacting pairs) and ArI + 2° alcohol couplings (two unique reacting pairs), dioxane and DMAc are favoured, respectively. For ArBr + 1° alcohol’s reactome (two unique reacting pairs), these two solvents are revealed to have less importance. In fact, the solvent of importance, allyl alcohol, is also the nucleophile in these couplings. In this subreactome, ligand identity plays a large role in yield determination. Finally, for the ArI + 1° alcohol’s reactome, reaction temperature is a leading factor, a point that is of no surprise^40^. It was expected to also see some importance placed on temperature for the other three subreactomes, but due to HTE design, temperature remained nearly constant throughout the entire subreactome, eliminating it as a variable.

For the Ullmann dataset, we believe the HTE and literature reactomes are in broad agreement. Gratifyingly, phenanthroline-based and picolinamide-based ligands are present in the best-in-class ligands, which are well known as privileged scaffolds in Ullmann couplings^41,42^. HiTEA observed that the Ma ligands (DMPAO and PMPBO) were individually less successful than other ligands in the standardized format of this HTE dataset. These ligands are characterized by high yields in the literature, which acknowledges that their yields are sensitive to the electronics of the specific ligand-reactant pairing^43^. Thus, it is possible that the true potential of these ligands were masked. Visualization of the ligand space reveals a very narrow scope for ligand choice, perhaps unsurprisingly given the similarity of privileged scaffolds in Ullmann couplings (Fig. 4a and Supplementary Fig. 14). A unique observation for the Ullmann’s ligand PCA is that the clustering is confined to the best-in-class ligands, supporting the random forest, *z*-score–ANOVA–Tukey findings that, for the most part, a few select ligands are useful for good yield outcomes. This outcome highlights the sensitivity of HiTEA’s best- and worst-in-class catalyst analysis: despite the similarity in structure of the ligands, key differences in performance were identified, leading to a remarkably subtle overall ranking. The selection of specific solvents within the subreactomes was also intriguing. Although all the solvents identified are known to be good solvents within the literature, it is striking how each solvent’s importance varies across subreactomes. Solvent effects are known to play a role in the mechanism of Ullmann couplings, but an exact understanding of which solvents are best for single electron transfer (SET) versus iodo-atom transfer (IAT) or for C–N versus C–O coupling is not fully characterized, despite observed preferences^40,44,45^. A deeper dive into solvent characteristics is recommended for a more comprehensive understanding of this reactome overall.

## Hydrogenations

Hydrogenations are a well utilized reaction with a broad range of applications^46,47^. The mechanistic differences between heterogeneous and homogeneous hydrogenations warrant that these datasets be analysed separately. Similar to the Buchwald–Hartwig dataset, the heterogeneous hydrogenations sample the reaction space in a ‘wide but shallow’ manner, whereas the homogeneous hydrogenations follow the Ullmann’s ‘narrow but deep’ scope. The overall diversity of the molecules for both hetero- and homogeneous hydrogenations are broad (Supplementary Fig. 13). We will not be delving deeply into the heterogeneous reduction of ‘other Functional groups (FGs) (those which contain a mixture of nitro, diazo and nitrile reductions) nor the heterogeneous alkyne reductions due to this reaction type containing only a single unique molecule undergoing hydrogenation (Fig. 2c,d).

**Fig. 5:**
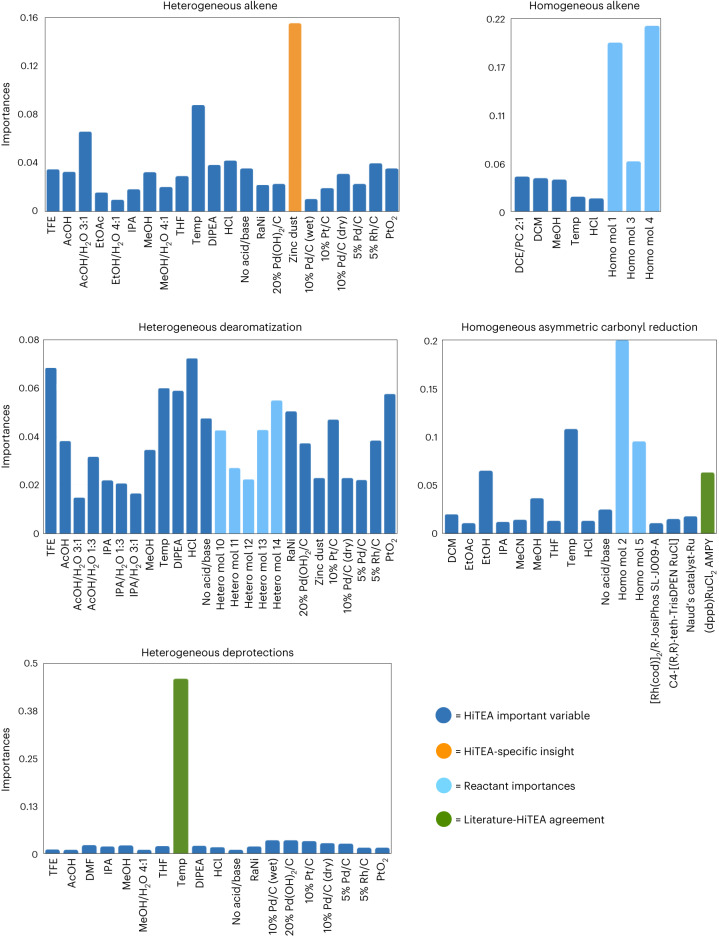
HiTEA variable importance analysis on heterogeneous and homogeneous hydrogenation datasets. Where appropriate, reactant importances are shown. HiTEA-specific variable importances are highlighted, as well as agreement between the literature and HiTEA variable importances. Acronym structures can be found in Supplementary Fig. 11.

**Fig. 6:**
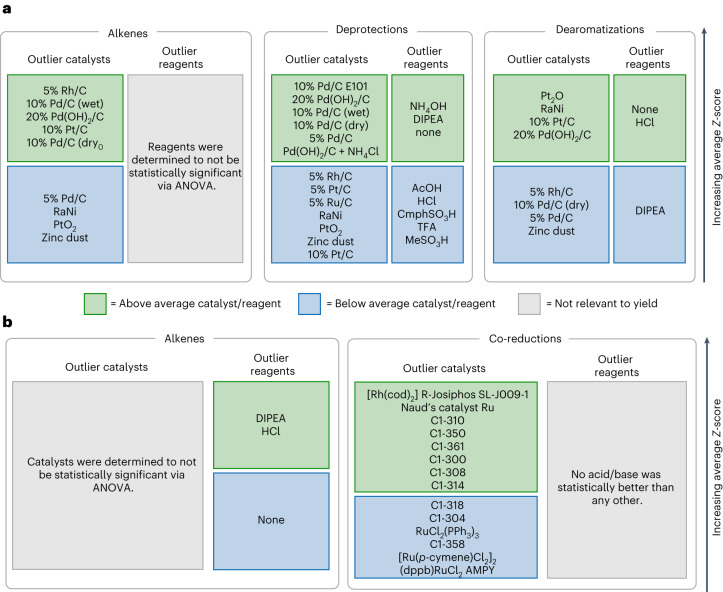
HiTEA best-/worst-in-class analysis of hydrogenation dataset. Acronym structures can be found in Supplementary Fig. 11. **a**, Heterogeneous hydrogenation dataset is shown. **b**, Homogeneous hydrogenation dataset is shown.

HiTEA reveals that the heterogeneous alkene subreactome (three unique reactants) places high negative importance on zinc dust, and for the HTE deprotection subreactome (nine unique reactants), a high positive importance on temperature (Figs. 5 and 6). While temperature-correlated deprotections do agree with the literature’s reactome^48,49^, the negative correlation with zinc dust is a HiTEA-specific insight. This exemplifies the value of the negative results in the dataset, which enables HiTEA to confirm negative correlations. The literature’s reactome is often unable to confirm such correlations as it lacks publications with the negative data required. Interestingly, the other three subreactomes have no standout variable. In the case of the homogeneous hydrogenations (11 unique reactants), this can be explained by a strong dependence upon the reactants, but dearomatizations (five unique reactants) show little overall dependence upon any variable, including molecule identity, perhaps due to the diverse and subtle changes that govern the energetically demanding process of dearomatization (Fig. 5)(ref. ^50^). For these three subdatasets, the HiTEA’s best-/worst-in-reaction-type reveal more information (Fig. 6).

Overall, higher loadings of Pd/C are better than lower loadings, and Pearlman’s catalyst is an all-around good catalyst for heterogeneous hydrogenations, two observations that are mirrored in the literature’s reactome^51^. The pH of hydrogenolysis deprotections have been reported to have a marked effect in selectivity of the reaction, although acidic conditions are usually preferential^52^. Dearomatizations, primarily performed on nitrogen-containing heterocycles in this dataset, are partial to acidic conditions^53^. For the yield of asymmetric carbonyl reductions (six unique reactants), ligand structure was a key factor, with very little importance placed on the base. In the literature, preferential treatment is given to rigid-backboned ligands, as it is hypothesized that flexible backbones deform the chiral pocket, leading to lower stereoselectivities^54,55^. Indeed, even yield in this subreactome is completely dominated by ligand structure, with the top performing ligand featuring a rigid six-member ruthenium metallocycle of a Josiphos ligand. The middling ligands contain either seven- or eight-member metallocycles. The poorest performer is (dppb)RuCl_2_AMPY, which boasts a more flexible backbone from the rotational bonds between the P–P bridge (Fig. 6)^56^. Once again, ligand visualization reveals pockets of best- and worst-in-class ligand scaffolds, with clear distinctions between the best of the best-in-class ((*R*)-Josiphos SL J009-1 and Naud’s catalyst Ru; see Supplementary Fig. 11 for all structures of acronyms) and the worst of the worst-in-class (*p*-cymene and dppb/AMPY), further supporting our chemical understanding of ligand design in metal-mediated asymmetric carbonyl reductions. Gratifyingly, even among the very structurally similar C1-3## family of catalysts from Johnson Matthey, a noticeable delineation between the good and poor performers is visible (Fig. 4a). Finally, homogeneous alkene hydrogenations’ best-/worst-in-class analysis confirms its variable importances conclusions: across the subreactome as a whole, the choice of catalyst is not statistically relevant in the determination of yield. This is no doubt a case of dataset bias as all of HiTEA’s techniques failed to produce reasonable results: the random forest had low out of bag accuracy, the resulting random forest importances not including any catalysts, and the ANOVA deeming the catalysts as not statistically significant. A broader selection of alkene substrates and catalysts, or a subset of this dataset with less noise, would probably improve the utility of this subdataset.

## Applications of HiTEA

The hidden chemical insights brought to light by HiTEA have a multitude of potential applications. Three possible scenarios for HiTEA application are focused HTE, mechanistic interrogation and machine learning data preparation, which would be valuable for synthetic chemists and data scientists.

## Mechanistic interrogation

Keen understanding of the underlying reaction mechanism is advantageous for reaction optimization, and oftentimes, a deep understanding of a mechanism can lead to the development of new reactions and catalysts. However, many reaction mechanisms have seen only partial elucidation, especially those that feature organometallic transition states^57^. We imagine that HiTEA could identify hidden correlations between reaction inputs and measured reaction outcome, providing statistically robust evidence for or against mechanistic hypotheses. In the course of our manuscript, we discovered that solvent identity plays a substantial role in the yield of Ullmann couplings; however, unlike their Buchwald counterparts, the effect of solvent polarity on the multitude of potential halogen-atom transfer / single electron transfer catalyst intermediates has not been elucidated. As HiTEA has been designed to be applicable in even low data environments, it has conceivable utility in the investigations of other reaction mechanisms with limited screening.

## Bias identification for machine learning

Bias is detrimental to machine learning because it allows the model to ‘cheat’, relying on spurious correlations to get the right answer and leading to a lack of generalizability^58^. Take, for example, an image classification network recognizing a lion based on the savannah background rather than the animal’s own features^59^. Image classifiers now employ a variety of techniques to try to combat bias of this type in addition to using huge image datasets that will have images of their subjects in a variety of backgrounds, poses and distances.

For chemistry, HTE data has been noted as a valuable source of data for machine learning algorithms, as it is one of the best ways to generate moderate-to-large scale amounts of data in a parallel fashion. However, these data will also have some bias: the reagents chosen by the chemist running the screen, the reaction is known to fail with specific motifs thus those motifs are left out of the dataset, or the simple fact that HTE is limited to the set of synthesizable molecules, which can be thought of as a bias, albeit one that we may want the network to learn or to operate in. As observed in the previous sections, HiTEA is adept at finding areas of bias in datasets, which usually take the form of substrate bias. When using these biased datasets for machine learning, one can either (1) augment the dataset with further rounds of HTE or additional datasets or (2) take a subset of the dataset that is less noisy and less biased; a removal of outliers. Both tasks can be aided by HiTEA through iterative augmentations or reductions followed by HiTEA. Stable and chemically sound HiTEA results (the removal of the surprising insights) indicate a dataset that is relatively robust and superior for consistent modelling.

## HiTEA for future HTE screens

The most straightforward application of HiTEA is for future reaction optimization reactions, either in high-throughput or in batch. While HTE can explore swaths of chemical space, the combinatorial cross of all possible reagents × catalysts × ligands × additives with even a limited set of reactants is unfeasible. HiTEA can give a visualization of the breadth of the scope and rapidly assess the statistically significant best and worst reagents, guiding the chemist to optimal reaction outcome. One could imagine HiTEA being used in conjunction with Shields et al.‘s Bayesian optimizer for even faster optimization^60^. Additionally, temporal analysis is straightforward to run to visualize trends in poor and excellent conditions over time, adding further versatility to HiTEA’s utility in reaction screening.

## Conclusions

Dataset exploration is an overlooked but critical area of research in data-driven chemistry. The experimentalist is often blind to the chemical insights that have been locked into these datasets, missing key directions towards areas of exploration. With the development of HiTEA, a meaningful step in addressing this challenge has been made. We uncovered several interesting areas of exploration within Ullmann reactomes and identified several reactomes, which would mostly benefit from additional HTE. We hope that this publication serves as a call to arms to the chemical community to collect, publish and analyse additional chemistry HTE data, providing further opportunities to explore the uncharted territories of the chemical reactome.

## Methods

### Materials

The random forest analysis was performed with Scikit’s sklearn.en-semble.RandomForestRegressor() (Scikit version 1.0.1) with python 3.7. Canonicalization of the molecule SMILES strings was performed with rdkit (version 2020.09.01). Visualization of the Tanimoto squares was performed with matplotlib.pyplot() version 3.3.4. Morgan fingerprints were formed with rdkit’s Chem.rdMolDescriptors.GetMorganFingerprintAsBitVect() using 2,048 bits. PCA visualization of the ligand Morgan fingerprints was performed with sklearn.decomposition.PCA(). All other analyses were performed in R (version 3.4.4) with RStudio as the integrated development environment. However, corresponding python code for all R analyses has been provided in the GitHub repository^61^. Correlations were performed with R’s built-in cor() function; ANOVA and Tukey tests were run with aov() and TukeyHSD(), respectively. One-hot encoding was achieved with the mltools library’s one_hot() function. All data manipulation in R was performed on data tables, with the data.table library.

### Example dataset cleanup

Reactions with missing temperature entries were removed. Duplicated rows, duplicated reagents and nonsensical reagents were also removed. Reactions whose profile did not fit the standard reaction profile (for example, no catalyst, no base, unusual substrates or reagents identified via visual inspection) were flagged for manual evaluation and corrected or discarded as necessary. Reactants were then split into their reacting components (for example, nucleophile/electrophile). Manual inspection of any outliers was always performed to confirm correct sorting. If catalysts were present, metal sources were separated from ligands. Each reagent, reactive substrate(s) and catalyst–ligand pair were then one-hot encoded and checked for correlation. Any variable with a correlation of 85% or higher was combined with its correlated variable.

## Online content

Any methods, additional references, Nature Portfolio reporting summaries, source data, extended data, supplementary information, acknowledgements, peer review information; details of author contributions and competing interests; and statements of data and code availability are available at 10.1038/s41557-023-01393-w.

### Supplementary information

Supplementary Information Materials and methods, including supplementary discussion and Supplementary Table 1 and Figs. 1–14.

## Data Availability

Further details of the analysis is available in the supplementary materials. The full dataset is available for download at our corresponding GitHub repository (https://github.com/emmaking-smith/HiTEA).

## Code availability

Code and datasets can be found at https://github.com/emmaking-smith/HiTEA.
