# Supplementary material for: Probing the chemical ‘reactome’ with high-throughput experimentation data
Source: Nat Chem. 2024 Jan 2;16(4):633–43. doi: 10.1038/s41557-023-01393-w (PMC10997498; doi:10.1038/s41557-023-01393-w)
Supplement: Supplementary file 1 — Materials and methods, including supplementary discussion and Supplementary Table 1 and Figs. 1–14. [file 41557_2023_1393_MOESM1_ESM.pdf]

nature chemistry

Article

https://doi.org/10.1038/s41557-023-01393-w

Probing the chemical 'reactome' with high-throughput experimentation data

In the format provided by the authors and unedited

Table of Contents

Materials and Methods.. page S2
Table S1: Random Forest out of bag (OOB) accuracy for each of the reaction classes.. page S5
Figure S1.. page S6
Figure S2.. page S7
Figure S3.. page S8
Figure S4.. page S9
Figure S5.. page S10
Figure S6.. page S11
Figure S7.. page S12
Figure S8.. page S13
Figure S9.. page S14
Figure S10.. page S15
Figure S11.. page S16
Figure S12.. page S17
Figure S13.. page S18
Figure S14.. page S19

Materials and Methods

The random forest analysis was performed with Scikit's sklearn.ensemble.RandomForestRegressor() (Scikit version 1.0.1) with python 3.7. Canonicalization of the molecule SMILES strings was performed with rdkit (version 2020.09.01). Visualization of the Tanimoto squares was performed with matplotlib.pyplot() version 3.3.4. Morgan fingerprints were formed with rdkit's Chem.rdMolDescriptors.GetMorganFingerprintAsBitVect() using 2,048 bits. PCA visualization of the ligand Morgan fingerprints was performed with sklearn.decomposition.PCA(). All other analyses were performed in R (version 3.4.4) with RStudio as the integrated development environment. However, corresponding python code for all R analyses has been provided in the GitHub repository (https://github.com/emmaking-smith/HiTEA). Correlations were performed with R's built-in cor() function, ANOVA and Tukey tests were run with aov() and TukeyHSD(), respectively. One-hot encoding was achieved with the mltools library's one_hot() function. All data manipulation in R was performed on data tables, with the data.table library.

Data Cleanup:

Buchwald-Hartwig and Ullmann Couplings:

Reactions with missing temperature entries were removed. Duplicated rows, duplicated reagents, and nonsensical reagents were also removed. Reactions whose profile did not fit the standard Buchwald-Hartwig or Ullmann coupling (i.e. no palladium/copper catalyst, no base, unusual substrates identified via visual inspection), were flagged for manual evaluation and corrected or discarded as necessary. Reactants were then split into aryl halide and nucleophiles. If a compound had both an aryl halide and a nucleophile, the nucleophile took priority. Manual inspection of these outlier was always performed to confirm their correct sorting. Next, the catalysts were sorted. The dataset has four columns that describe the catalyst system, however, only two are relevant for the couplings. Palladium/copper sources (including pre-complexed metal sources) were separated from ligands. Each reagent, halide-nucleophile pair, and catalyst-ligand pair were then one-hot encoded and checked for correlation. Any variable with a correlation of 85% or higher was combined with its correlated variable.

Hydrogenations:

Reactions with missing temperature entries were removed. Duplicated rows, duplicated reagents, and nonsensical reagents were also removed. Reactions were split into heterogeneous and homogeneous reactions via their catalyst identity. The homogeneous reactions' catalysts were further sorted into metal source (including pre-complexed metals) were separated from ligands. Each reagent, halide-nucleophile pair, and catalyst-ligand pair were then one-hot encoded and checked for correlation. Any variable with a correlation of 85% or higher was combined with its correlated variable.

Example High Throughput Experimentation Procedure:

HTE reactions were set up inside of an INERT Inc. triple double sized glove box with O_2 and H_2O levels < 20 ppm. Glass vials (0.3 mL, 8 x 20 mm) pre-equipped with stir bars were used for each reaction except for chiral salt resolutions (0.7 mL, 8 x 30 mm vials). The reactions were set up with the components and conditions described by each dataset entry at 0.01M final

concentration of limiting reactant and 100 μL total volume, except for chiral salt resolutions (0.20M and 200 μL respectively). The reaction vials were sealed by crimp under the glove-box environment and placed in a metal Chemglass Optichem 96-well heating plate on top of a general IKA stirrer heater plate with an external temperature probe to accurately and evenly control the plate. In the case of pressure reactions, the 8 x 20mm crimped vials' septa were pierced with a 16G needle to enable gas ingress at pressure. The vials were removed from the glove-box and quickly inserted into the pressure apparatus and after three nitrogen purges, placed under 8 Bar of pressure and heated to the desired temperature for the set period. Other than the chiral salt resolutions, after the set period the reactions were cooled, diluted with acetonitrile (200 μL) or DMF (200 μL) depending on solubility and to ensure dissolution, mixed, centrifuged, and directly analyzed by UPLC-MS. UPLC-MS analysis used a Waters Acquity UPLC BEH, C18 17 μm 2.1 x 30 mm column with A: 0.1% AcOH/NH_4COOH/H_2O, B: MeCN / 0.1% AcOH/NH_4COOH/H_2O linear gradient over 0.8 minutes running from 5% B/A to 95% B/A at 100˚C and a flow rate of 2.5 mL / min with a UV detection wavelength of 210 - 360 nm. Injections (0.5 μL) were made directly from diluted reaction mixtures and ionization monitored in positive mode scanning from 100 to 800 MW and with an additional SIM channel specifically for the ion of interest. For chiral salt resolutions, at the reaction end point the vials were centrifuged and the liquors sampled and analyzed by chiral SFC-MS (individual methods unavailable). For any enriched liquor hits, the liquor was manually pipetted away from the centrifuged solid and the solid re-slurried in the reaction solvent (half volume, 100 μL). The vial was centrifuged a second time and the liquor (100 μL) again removed from the centrifuged solid and combined with the original. Both the combined liquors and residual solid where brought up to an equal volume using MeOH (500 μL) to ensure solution before being analyzed by chiral SFC-MS. Measurement of the crystallized salt product %ee from isolated solids superseded the product %ee calculated based on SFC-MS analysis of uncrystallized material in the liquors. iChem explorer (Reaction Analytics, US) and Virscidian Analytical Studio™ software were used for data analysis.

Discussion of Yield Determinations:

The medicinal chemistry HTE dataset analyzed herein, "percent yield" from each reaction is an uncalibrated value, essentially percent area/area UV of desired product versus total integrated peaks (product, unreacted starting material and byproducts, as selected by the acquiring scientist). Often the limiting reactant and product have similar UV absorbance profiles, making this a fair representation of true percentage conversion. Furthermore, a correlation of UV area and product mass ion count has been made across each reaction screen to highlight and remove erroneous data, for instance due to overlapping peaks.

Random Forest Parameters:
The random forest regressor used to determine the variable importances was the default sklearn's ensemble.RandomForestRegressor(). The following parameters were used:

n_estimators = 100	**max_leaf_nodes = None**	verbose = 0
criterion = absolute_error	min_impurity_decrease = 0.0	warm_start = False
max_depth = None	bootstrap = True	ccp_alpha = 0.0
min_samples_split = 2	oob_score = False	max_samples = None
min_samples_leaf = 1	n_jobs = None	max_features = 1.0

min_weight_fraction_leaf = 0.0	random_state = None

Morgan Fingerprints:
The default settings of RDKit's Chem.rdMolDescriptors.GetMorganAsBitVect() were used:

radius = 2	fromAtoms = []	useFeatures = False
nBits = 2048	useChirality = False	bitInfo = None
invariants = []	useBondTypes = True	includeRedundantEnvironments = False

Table S1: Random Forest out of bag (OOB) accuracy for each of the reaction classes.

Reaction Class	OOB Accuracy
Buchwald ArBr + 2˚ amines	73%
Buchwald ArCl + 1˚ amines	76%
Buchwald ArI + 1˚ alcohols	12%
Buchwald ArBr + amides	65%
Buchwald ArBr + 1˚ amines	70%
Buchwald ArBr + 1˚ alcohols	8%
Ullmann ArI + aromatic N	89%
Ullmann ArI + 1˚ alcohols	100%
Ullmann ArI + 2˚ alcohols	66%
Ullmann ArBr + 1˚ alcohols	43%
Heterogeneous Alkene	58%
Heterogeneous Deprotection	89%
Heterogeneous Dearomatization	33%
Homogeneous Alkene	3%
Homogeneous CO reduction	22%

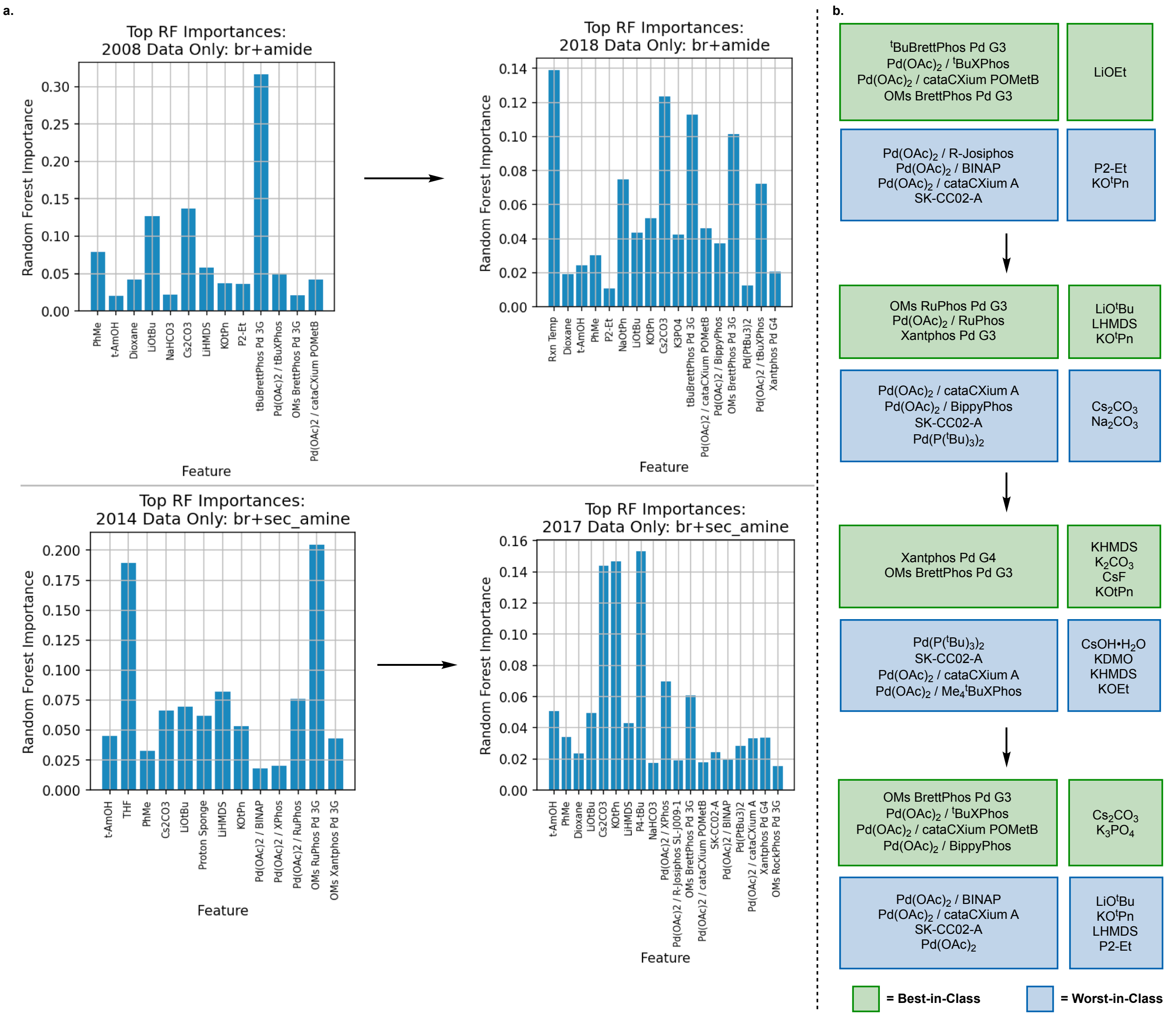

Fig. S1: Temporal analysis of Buchwald Hartwig reaction classes. Arrows indicate flow of time. Analysis performed on reaction classes with more than 100 reactions per year. **a.** Random forest importances. **b.** Overall best-in-class / worst-in-class catalysts and reagents.

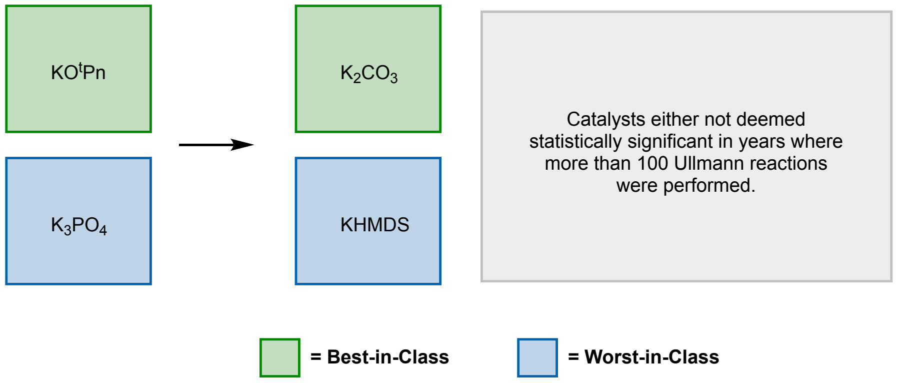

Fig. S2: Temporal analysis of Ullmann reaction classes. Arrows indicate flow of time. Analysis performed on reaction classes with more than 100 reactions per year. Note that exploration of random forest importances was not possible on this dataset as the four reaction classes discussed in the main text did not have any temporal evolution: the majority of each reaction class was run in a single year. Thus, overall best-in-class / worst-in-class reagents are shown.

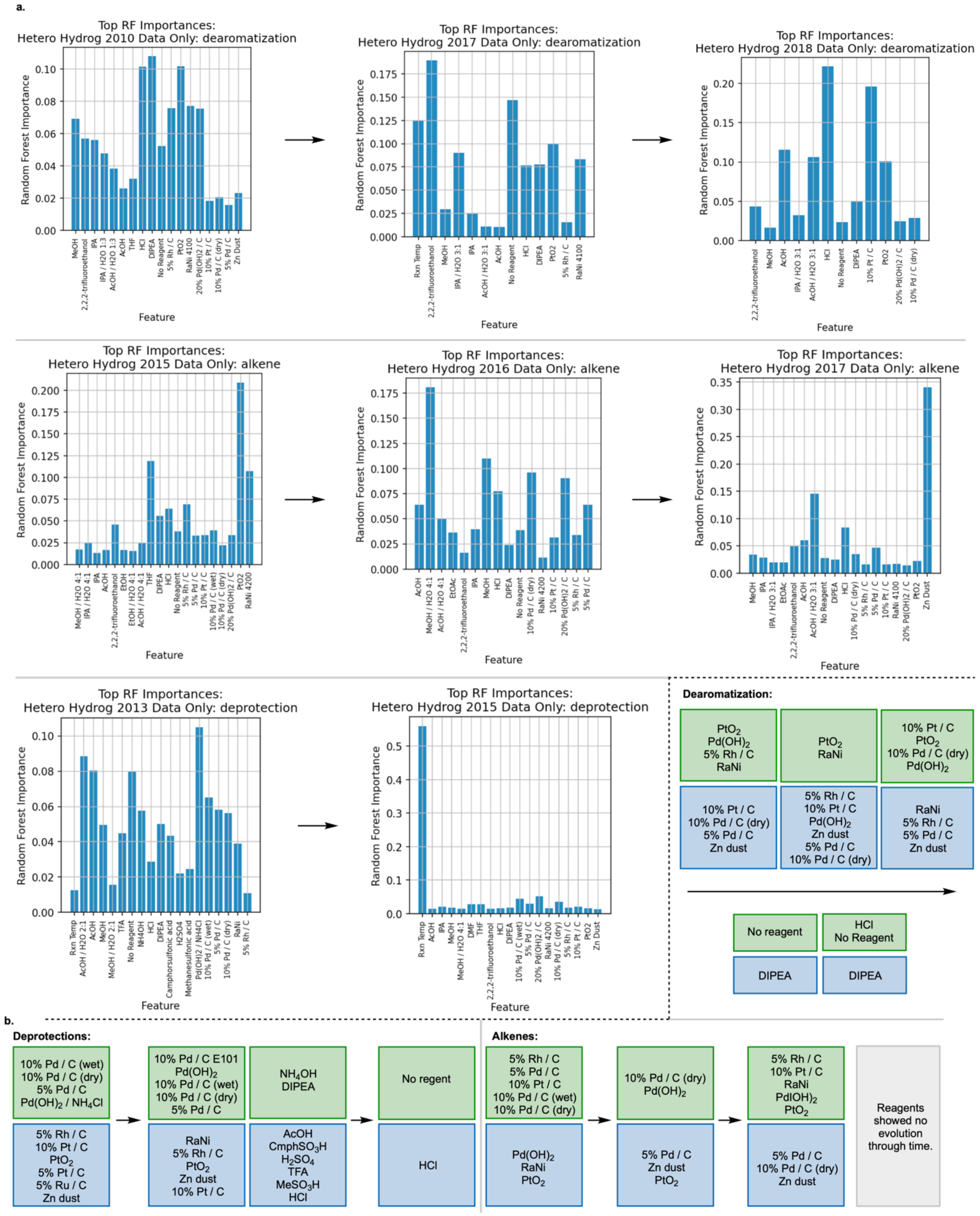

Fig. S3: Temporal analysis of heterogeneous hydrogenation reaction classes, and best-in-class / worst-in-class catalysts and reagents for each reaction class. Arrows indicate flow of time. Analysis performed on reaction classes with more than 100 reactions per year. **a.** Random forest importances. **b.** Overall (per reaction class) best-in-class / worst-in-class catalysts and reagents.

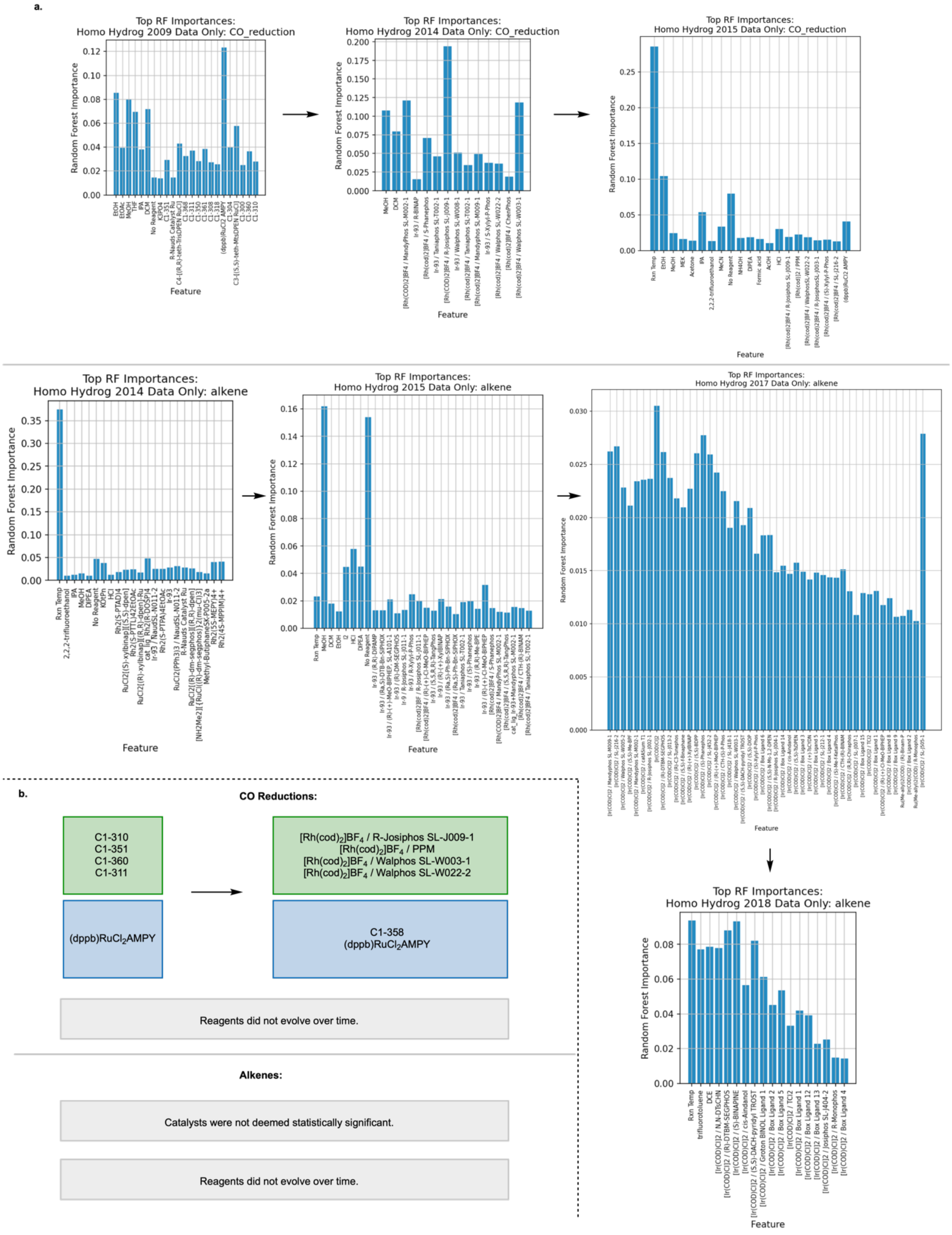

Fig. S4: Temporal analysis of homogeneous hydrogenation reaction classes, and best-in-class / worst-in-class catalysts and reagents for each reaction class. Arrows indicate flow of time. Analysis performed on reaction classes with more than 100 reactions per year. **a.** Random forest importances. **b.** Overall (per reaction class) best-in-class / worst-in-class catalysts and reagents.

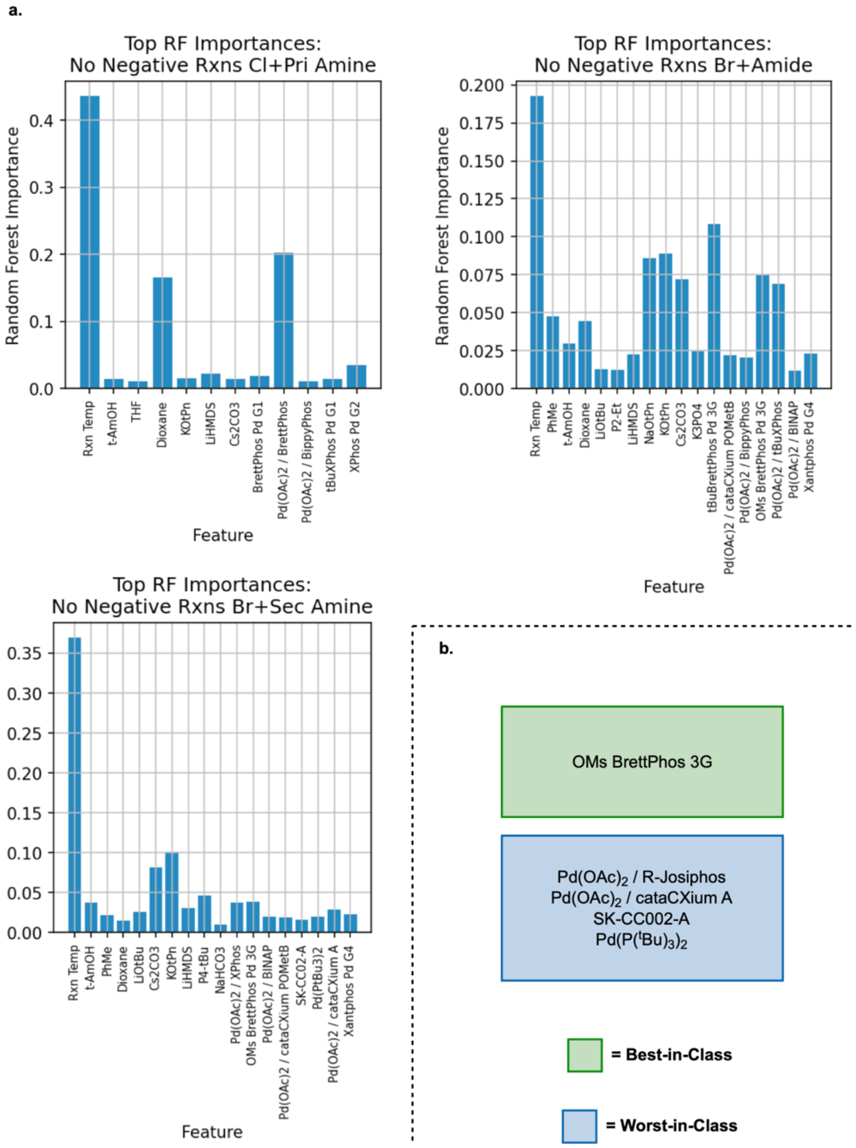

Fig. S5: Analysis of Buchwald Hartwig reaction classes upon removal of the 0% yielding reactions. Analysis performed on reaction classes with more than 100 reactions after removal of 0% yielding reactions. **a.** Random forest importances. **b.** Overall best-in-class / worst-in-class reagents.

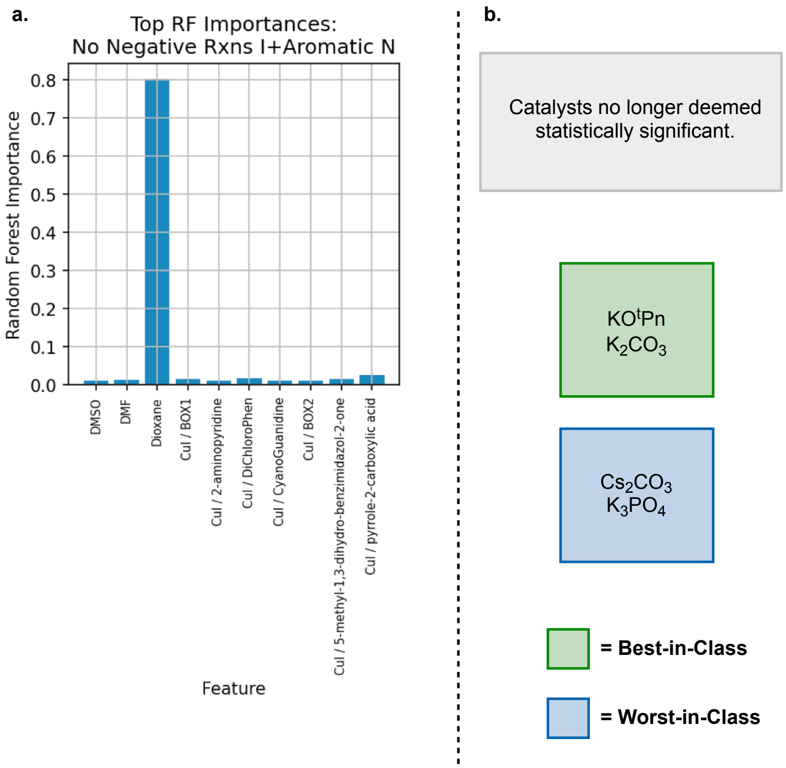

Fig. S6: Analysis of Ullmann reaction classes upon removal of the 0% yielding reactions. Analysis performed on reaction classes with more than 100 reactions after removal of 0% yielding reactions. **a.** Random forest importances. **b.** Overall best-in-class / worst-in-class reagents.

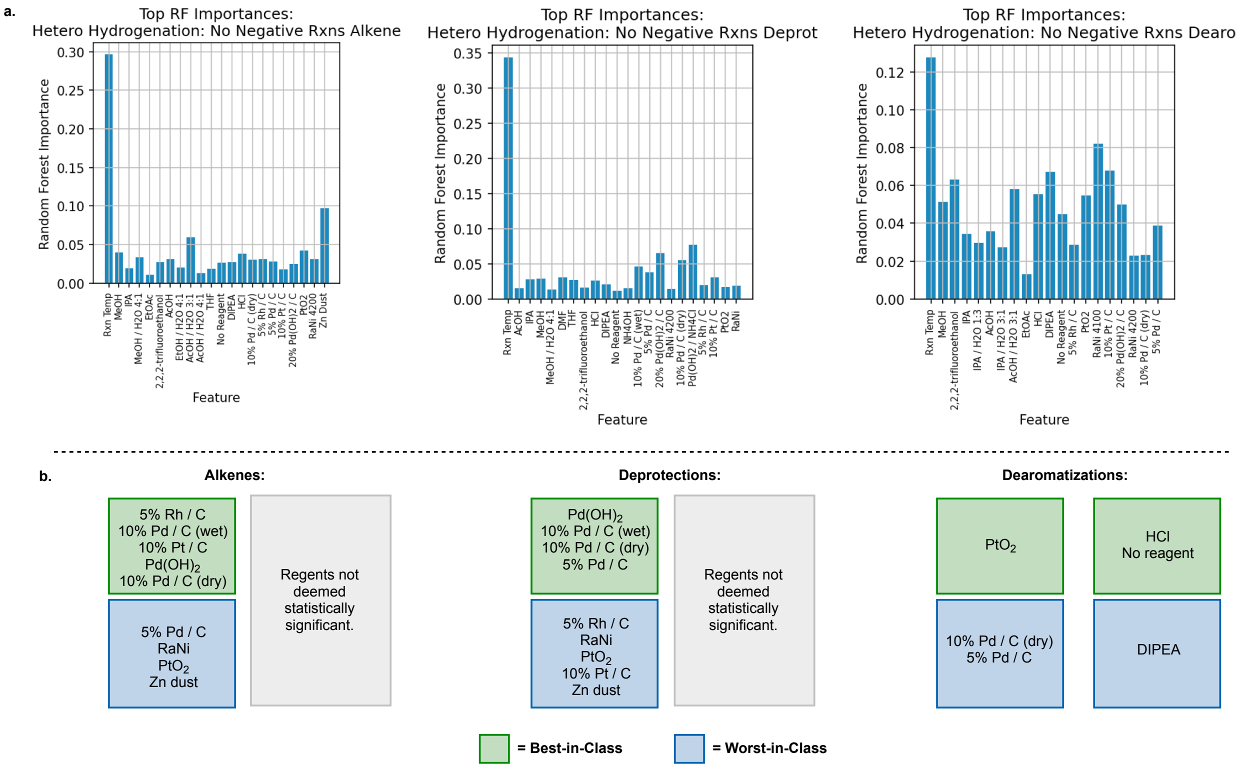

Fig. S7: Analysis of heterogeneous hydrogenation reaction classes upon removal of the 0% yielding reactions. Analysis performed on reaction classes with more than 100 reactions after removal of 0% yielding reactions. **a.** Random forest importances. **b.** Overall (per each reaction class) best-in-class / worst-in-class reagents.

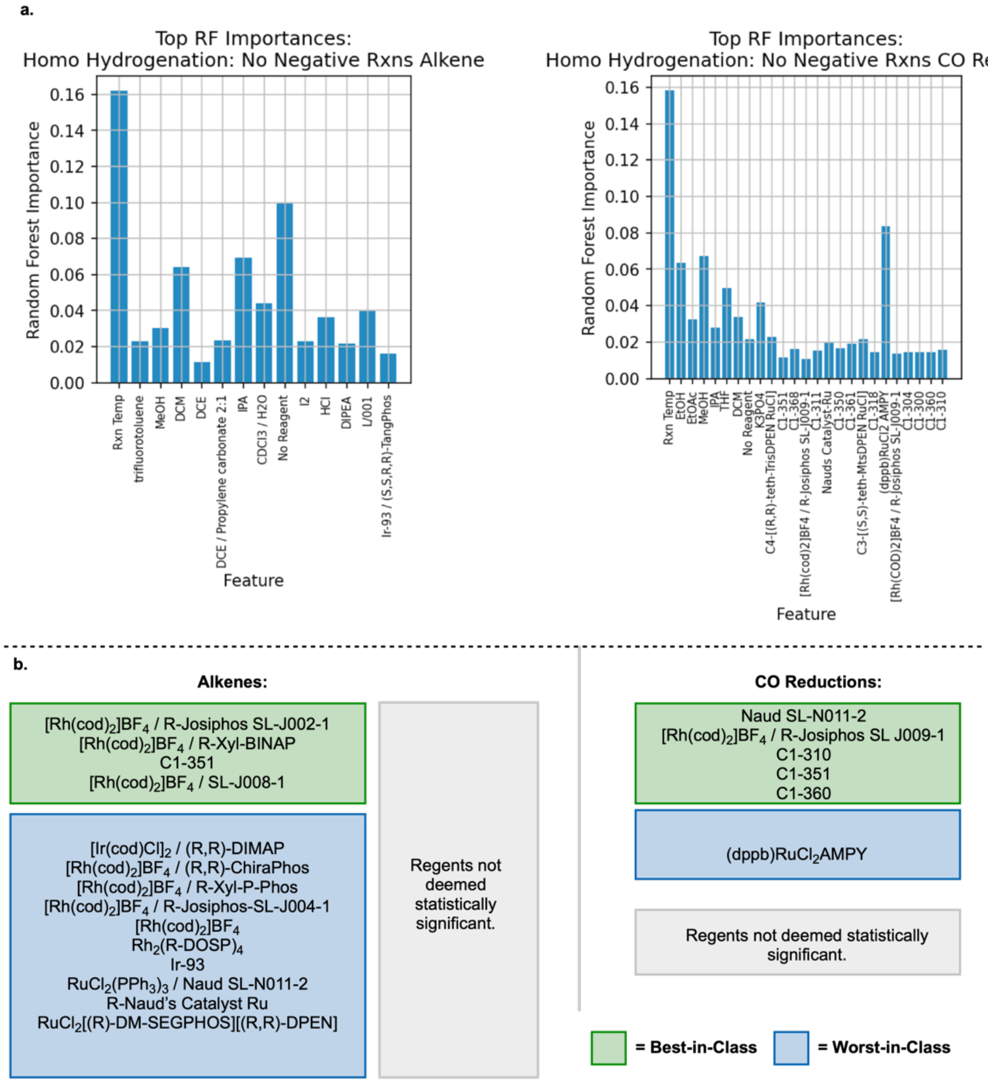

Fig. S8: Analysis of homogeneous hydrogenation reaction classes upon removal of the 0% yielding reactions. Analysis performed on reaction classes with more than 100 reactions after removal of 0% yielding reactions. **a.** Random forest importances. **b.** Overall (per each reaction class) best-in-class / worst-in-class reagents.

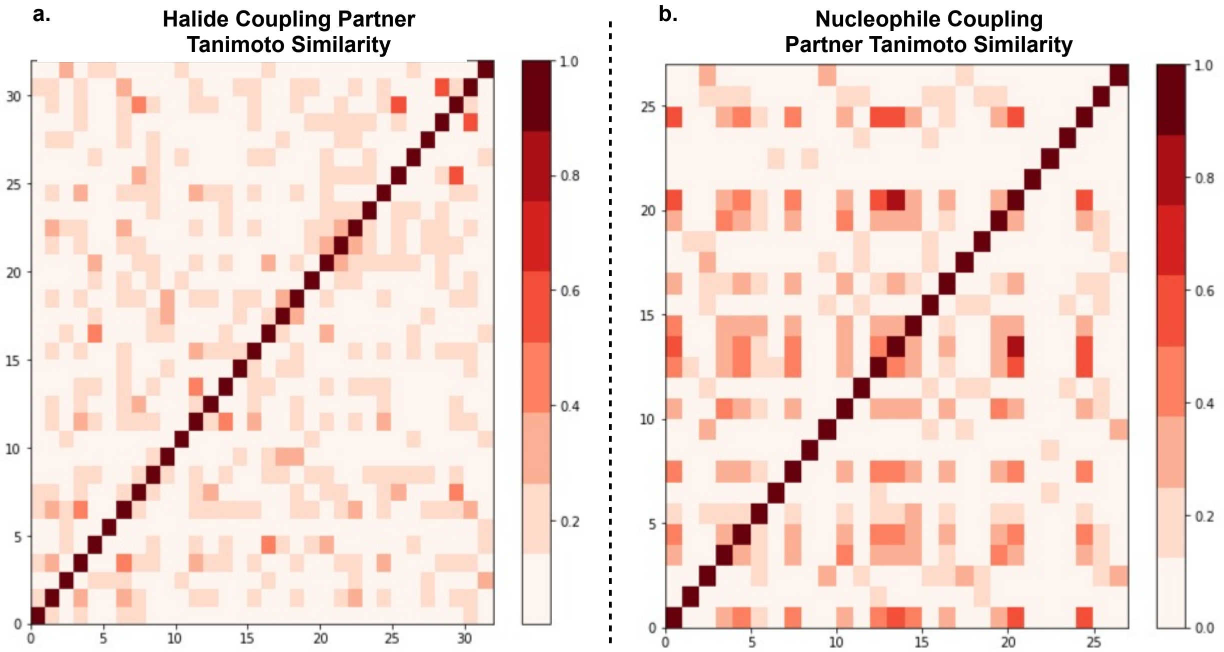

Fig. S9: Tanimoto similarity scores of the Buchwald-Hartwig coupling partners. Identical molecules are given a score of 1. **a.** Comparison of each halide coupling partner to every other halide in the dataset. **b.** Comparison of each nucleophile coupling partner to every other nucleophile in the dataset.

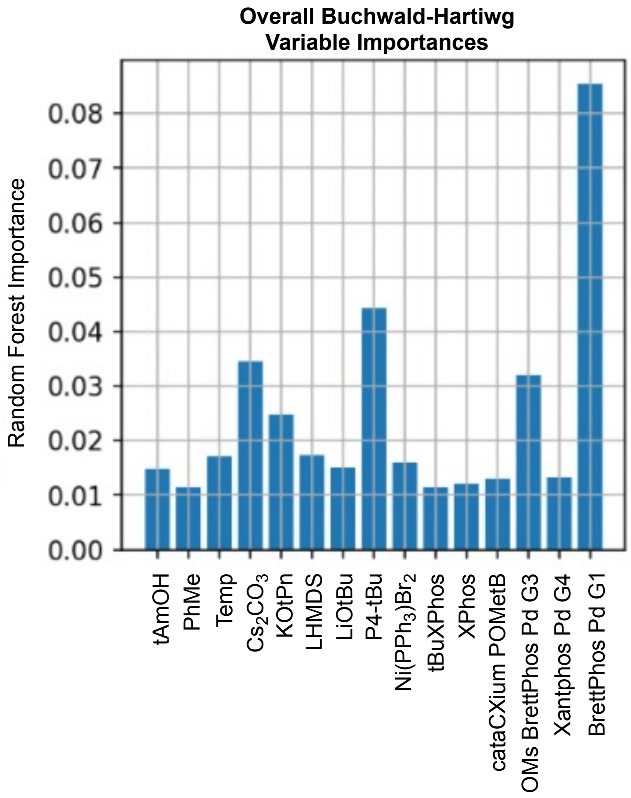

Fig. S10: The variable importances for the entire Buchwald-Hartwig dataset.

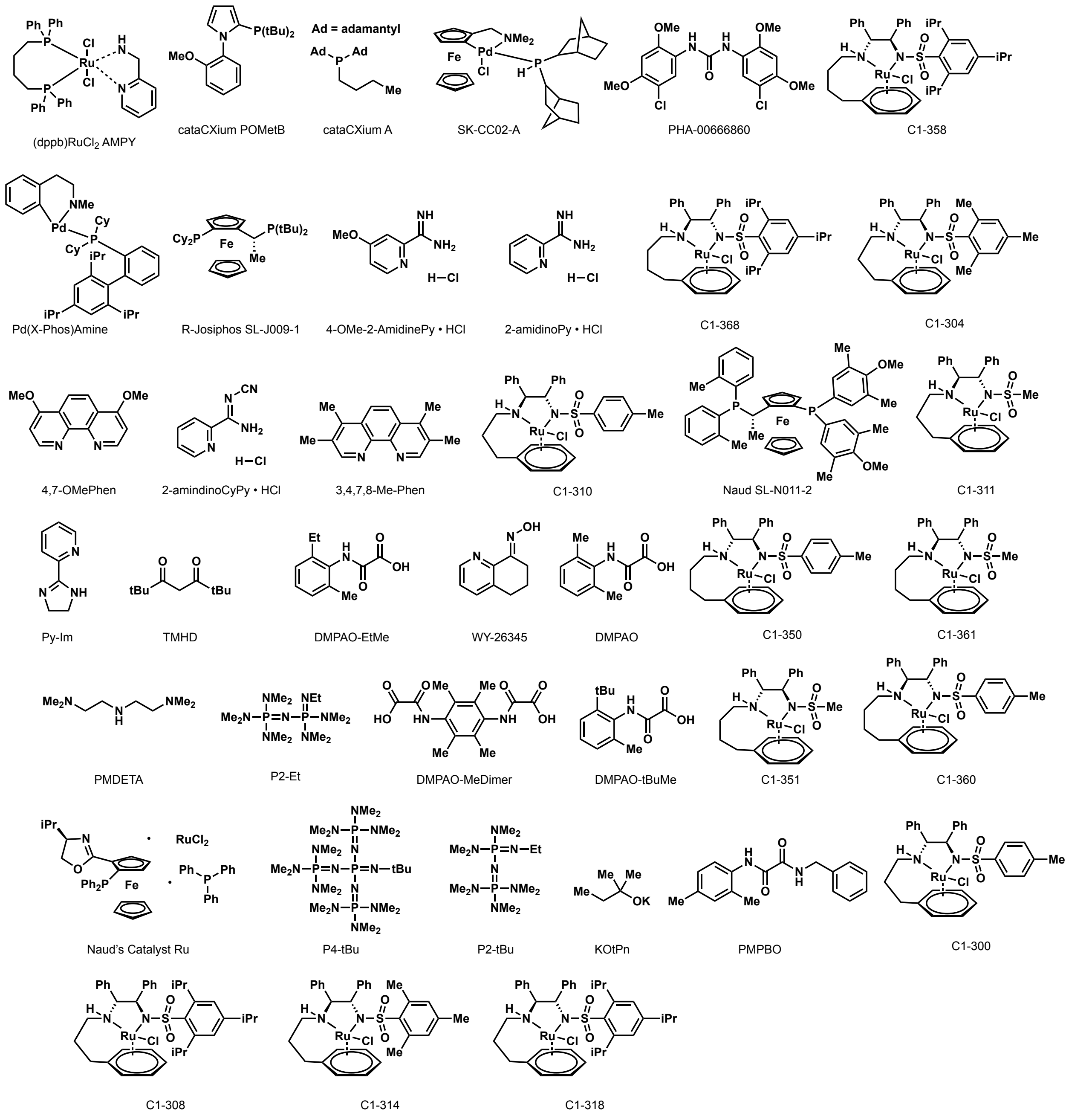

Fig. S11: The structures of the molecule acronyms present in the main text.

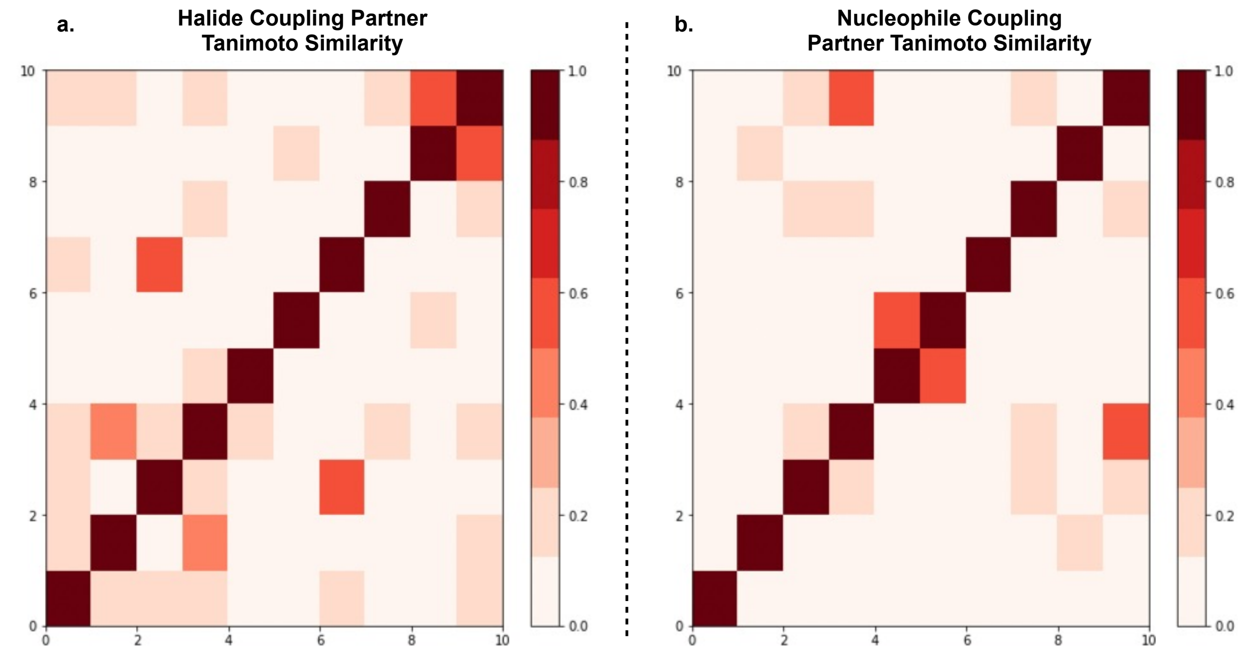

Fig. S12: Tanimoto similarity scores of the Ullmann coupling partners. Identical molecules are given a score of 1. **a.** Comparison of each halide coupling partner to every other halide in the dataset. **b.** Comparison of each nucleophile coupling partner to every other nucleophile in the dataset.

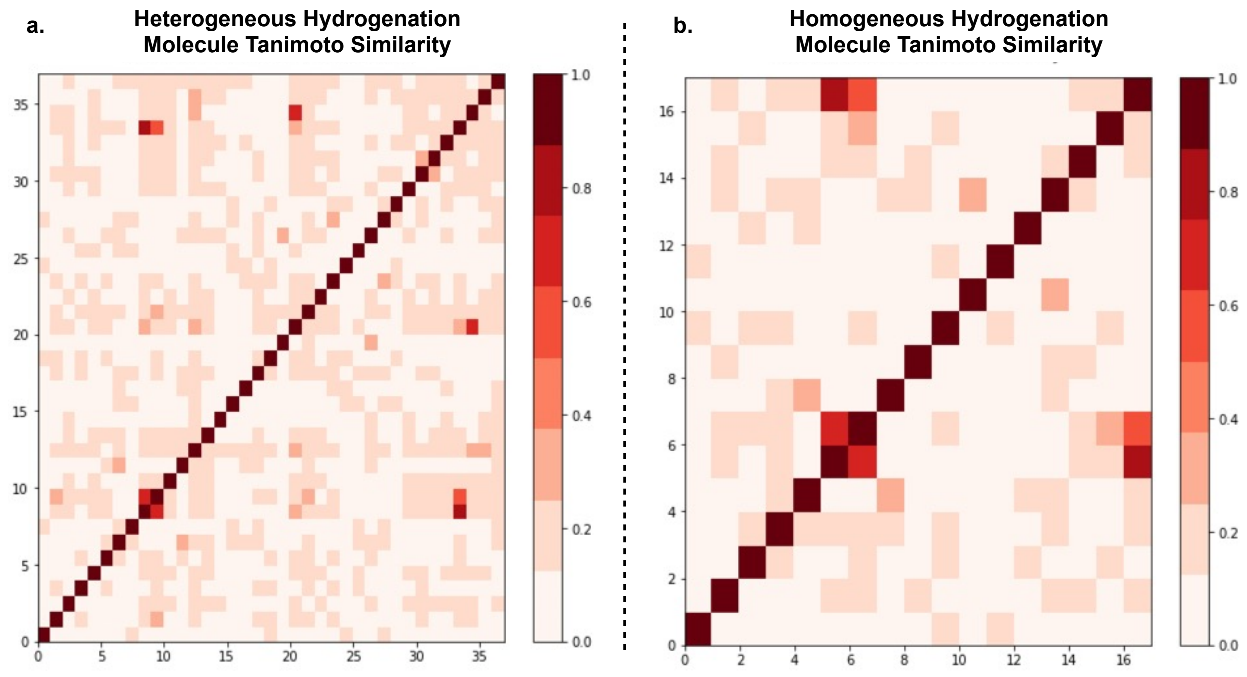

Fig. S13: Tanimoto similarity scores of the hydrogenation molecules. Identical molecules are given a score of 1. **a.** Comparison of each molecule to every other molecule undergoing heterogeneous hydrogenations. **b.** Comparison of each molecule to every other molecule undergoing homogeneous hydrogenations.

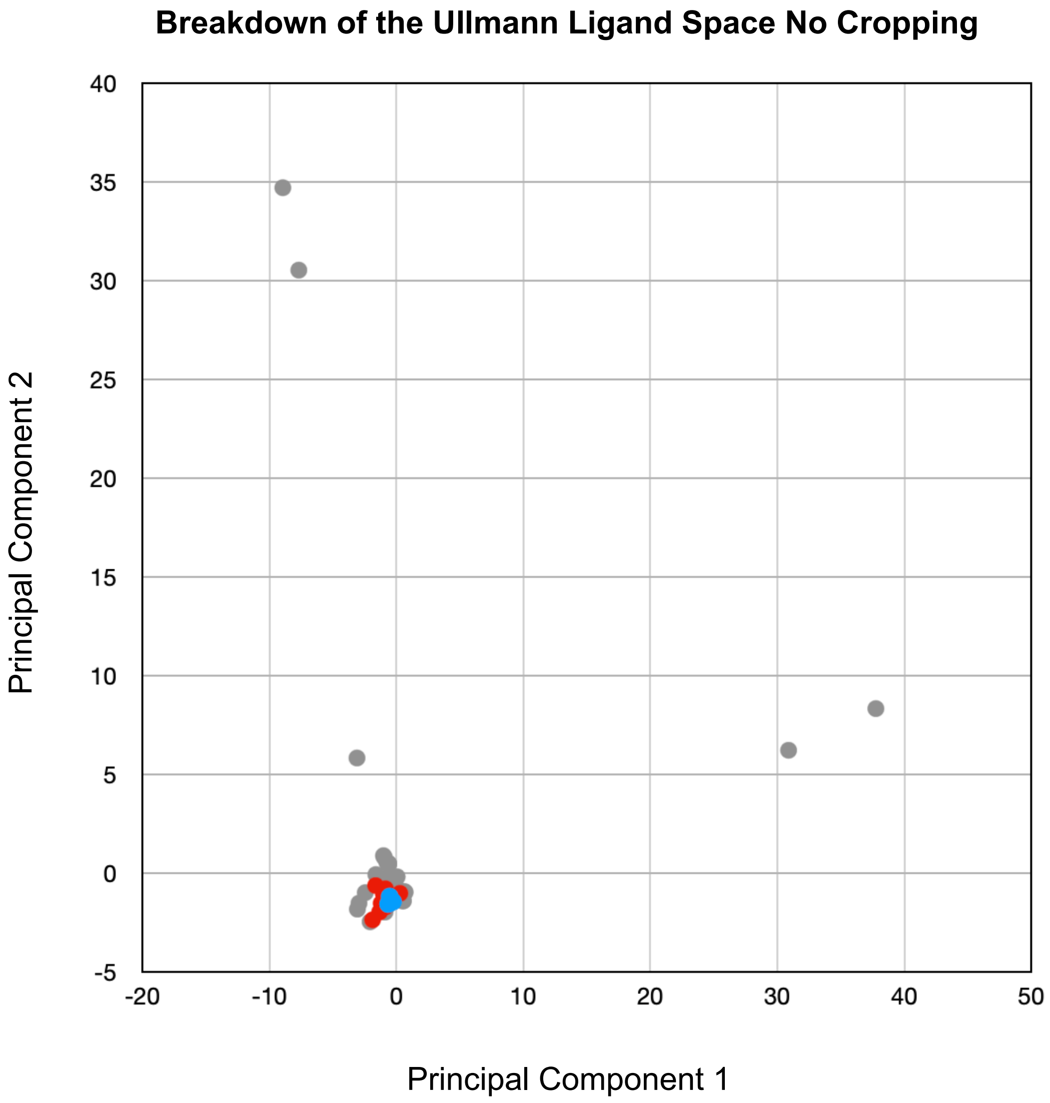

Fig. S14: The zoomed out PCA of the Ullmann ligands.
